# The effect of irrigation on malaria vector bionomics and transmission intensity in western Ethiopia

**DOI:** 10.1186/s13071-021-04993-y

**Published:** 2021-10-07

**Authors:** Werissaw Haileselassie, Endalew Zemene, Ming-Chieh Lee, Daibin Zhong, Guofa Zhou, Behailu Taye, Alemayehu Dagne, Wakgari Deressa, James W. Kazura, Guiyun Yan, Delenasaw Yewhalaw

**Affiliations:** 1 School of Public Health, College of Health Sciences, Addis Ababa University, Addis Ababa, Ethiopia; 2 School of Medical Laboratory Sciences, Institute of Health, Jimma University, Jimma, Ethiopia; 3 Program in Public Health, College of Health Sciences, University of California at Irvine, Irvine, CA 92697 USA; 4 Department of Biology, Faculty of Natural and Computational Science, Mettu University, Mettu, Ethiopia; 5 Center for Global Health and Disease, Case Western Reserve University, Cleveland, OH 44106 USA; 6 Tropical and Infectious Diseases Research Centre, Jimma University, Jimma, Ethiopia

**Keywords:** Mosquito vectors, Malaria, Transmission intensity, Irrigation, Ethiopia

## Abstract

**Background:**

Irrigation schemes may result in subsequent changes in malaria disease dynamics. Understanding the mechanisms and effects of irrigation on malaria vector bionomics and transmission intensity is essential to develop new or alternative surveillance and control strategies to reduce or control malaria risk. This study was designed to assess the effect of rice irrigation on malaria vector bionomics and transmission intensity in the Gambella Region, Ethiopia.

**Methods:**

Comparative cross-sectional study was conducted in Abobo District of the Gambella Region, Ethiopia. Accordingly, clusters (kebeles) were classified into nearby and faraway clusters depending on their proximity to the irrigation scheme. Adult mosquito survey was conducted in February, August and November 2018 from three nearby and three faraway clusters using Centers for Disease Control and Prevention (CDC) light traps (LTs). During the November survey, human landing catch (HLC) and pyrethrum spray catch (PSC) were also conducted. The collected mosquitoes were morphologically identified to species and tested for *Plasmodium* infection using circumsporozoite protein enzyme-linked immunosorbent assay (CSP-ELISA). Furthermore, species-specific polymerase chain reaction (PCR) was performed to identify member species of the *Anopheles gambiae* complex. Chi-square and *t*-tests were used to analyze the data using the SPSS version 20 software package.

**Results:**

A total of 4319 female anopheline mosquitoes comprising *An. gambiae* sensu lato, *An. funestus* group, *An. pharoensis*, *An. coustani* complex and *An. squamosus* were collected. Overall, 84.5% and 15.5% of the anopheline mosquitoes were collected from the nearby and faraway clusters, respectively. *Anopheles gambiae* s.l. was the predominant (56.2%) anopheline species in the area followed by *An. pharoensis* (15.7%). The density of anopheline mosquitoes was significantly higher in the nearby clusters in both HLCs [*t*_(3)_  =  5.14, *P*  =  0.0143] and CDC LT catches [*t*_(271.97)_  =  7.446, *P*  <  0.0001). The overall sporozoite rate of anopheline species from the nearby clusters was 10-fold higher compared to the faraway clusters.

**Conclusions:**

Significantly higher mosquito population density was observed in areas close to the irrigation sites. Sporozoite infection rate in the mosquito population was also markedly higher from the nearby clusters. Therefore, the irrigation scheme could increase the risk of malaria in the area.

**Graphical abstract:**

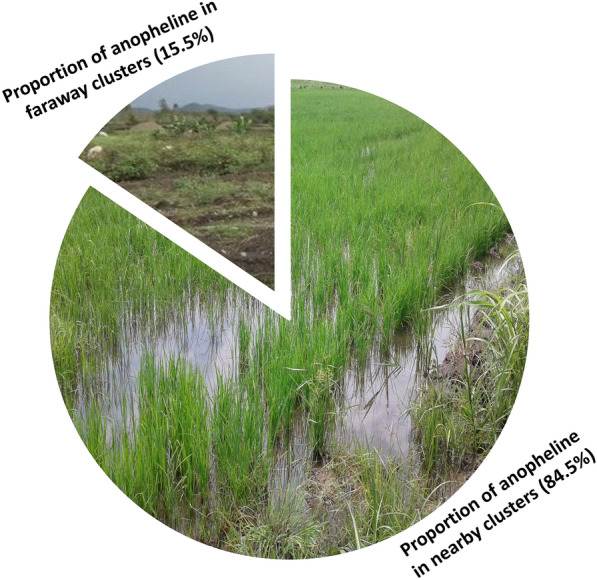

**Supplementary Information:**

The online version contains supplementary material available at 10.1186/s13071-021-04993-y.

## Background

Understanding bionomics of malaria vectors is pillar to develop and implement effective vector control interventions. Malaria transmission in Ethiopia varies spatio-temporally, mainly depending on microclimate and altitude [1]. In most parts of the country the transmission is seasonal and unstable. In the seasonal transmission areas, malaria cases usually peak following the major rainy season that extends from June to September. In these unstable transmission areas, there might also be a minor malaria transmission period that extends from April to June following minor rains in February and March. In the western lowlands, including Gambella Region, the transmission is generally high and year-round. While there is spatial variation in the distribution of the different species of anopheline mosquitoes in Ethiopia, *Anopheles arabiensis* is generally widely distributed and is the primary vector of malaria [2]. *Anopheles pharoensis*, *An. funestus* and *An. nili* are secondary vectors, while *An. coustani* is a suspected vector with limited distribution.

The dynamics of malaria transmission are affected by several factors including microclimate variation associated with altitude and ecological factors related to land-use changes. Ecological changes resulting from water resource development projects (WRDPs), including construction of hydroelectric dams and irrigation schemes, markedly affect the epidemiology of malaria [3, 4]. Irrigated rice cultivation in particular may provide favorable mosquito breeding habitats, resulting in higher vector density [5] and enhanced malaria transmission [6]. Rice irrigation schemes may create suitable larval ecology for *An. arabiensis,* which prefers open sunlit aquatic habitats [7]. However, higher vector mosquito density in rice irrigation areas may not always be translated to higher malaria transmission [5, 8]. Rice irrigation could also extend the duration of malaria transmission as a result of the presence of flooded fields suitable for mosquito breeding during the dry seasons [4]. Deforestation which may precede large-scale rice cultivation could also contribute to enhanced malaria transmission [9, 10], although the link between deforestation and malaria transmission is not always straightforward [11].

In Ethiopia, construction of irrigation projects and hydroelectric dams has been enhanced in recent years. It is obvious that such WRDPs are crucial to ensure food security and generate power, hence alleviating poverty [12, 13]. However, the possible ecological changes and the resulting impact on vector-borne diseases in general and malaria in particular is not adequately addressed in some of the WRDPs. Irrigated farming has the potential to create additional breeding sites enhancing malaria transmission [3, 14, 15]. Some studies have also documented increased malaria risk due to rice irrigation, mainly demonstrating its impact using entomological parameters [6, 16]. Rice irrigation may also alter the transmission dynamics of malaria from seasonal to perennial [17], likely as a result of prolonged presence of aquatic habitats suitable for mosquito breeding.

The effect of irrigation on risk of malaria appears to be complex. Ecological setting of the irrigation is one of the major factors affecting the epidemiology of malaria, with irrigation schemes undertaken in lowland areas remarkably favoring malaria transmission compared to those taking place in the highlands [18]. Moreover, the economic return from the irrigation scheme to the local community is another important factor affecting the net effect of irrigation on the risk of malaria. In some areas, reduced risk of malaria in irrigated areas has been reported [19, 20]. These factors dictate area-specific evaluation of impact of irrigation schemes on the risk of malaria transmission. Because of food insecurity issues, major investments in water resources are taking place in Ethiopia. One of the WRDPs is taking place in Gambella, where there is extensive rice cultivation through irrigation. As the rice farming in the area relies on flooded paddies, favorable mosquito breeding sites may be created, which has implications for the local community. In light of the aforementioned and anticipated expansion of WRDPs in Ethiopia in the years to come, understanding the effects of rice irrigation on malaria transmission dynamics in the area is crucial to effectively deploy malaria prevention. However, the potential effect of the land-use change due to irrigation on malaria transmission in the Gambella Region has not yet been studied. Therefore, the current study was designed to assess the effect of rice irrigation schemes on malaria vector bionomics and transmission intensity in the Gambella Region, Ethiopia.

## Methods

### Study setting

The study was conducted in Abobo District of Gambella Regional State, western Ethiopia. The district is located 811 km west of the capital, Addis Ababa. It is one of the districts in Anuak Zone of Gambella Regional State. The projected total population size of the district in 2019 was estimated to be 26,080 [21]. The total number of households in the district is 5670. The district is located at 7°51′0″ N, 34°33′0″ E, with altitude ranging from 500 to 700 m above sea level, and covers an area of 3116 km^2^. The area is characterized by savanna grassland, wetlands, and some portion covered by forest. The weather condition is hot (mean annual temperature range 28–37 °C) and humid with seasonal rainfall (annual rainfall range 900–1200 mm). The mean annual relative humidity of the district ranges from 74.1 to 88.3%. The hot and humid conditions, coupled with seasonal rainfall, create a favorable environment for mosquito breeding. The main socioeconomic activities of the community are farming and fishing. Cotton, maize, sorghum and fruits (mango, papaya and banana) are mainly grown by the local community. Fishing takes place in Alwero Dam, which is also used for large-scale irrigation of rice (owned by Saudi Star Agricultural Development PLC, a privately owned company). The canal extends 25 km from Alwero Dam, which is the major water body in the district, covering a total of 2700 hectares. The irrigation project was established in 2012 and has about 2000 workers. The current rice irrigation area is 3000 hectares, with planned expansion to 10,000 hectares. There has been extensive deforestation prior to rice cultivation. The irrigation scheme uses surface irrigation method. There is no crop rotation in the rice irrigation to avoid mixing of varieties, as there is a seed bank. While the irrigation farm workers live mainly in the Ghulam Rasool Company (GRC) and Bravo camps of the company, most of the residents in the district live in traditional houses constructed of wood and mud walls, with thatched roofs, and some live in houses with corrugated iron sheets.

**Fig. 1:**
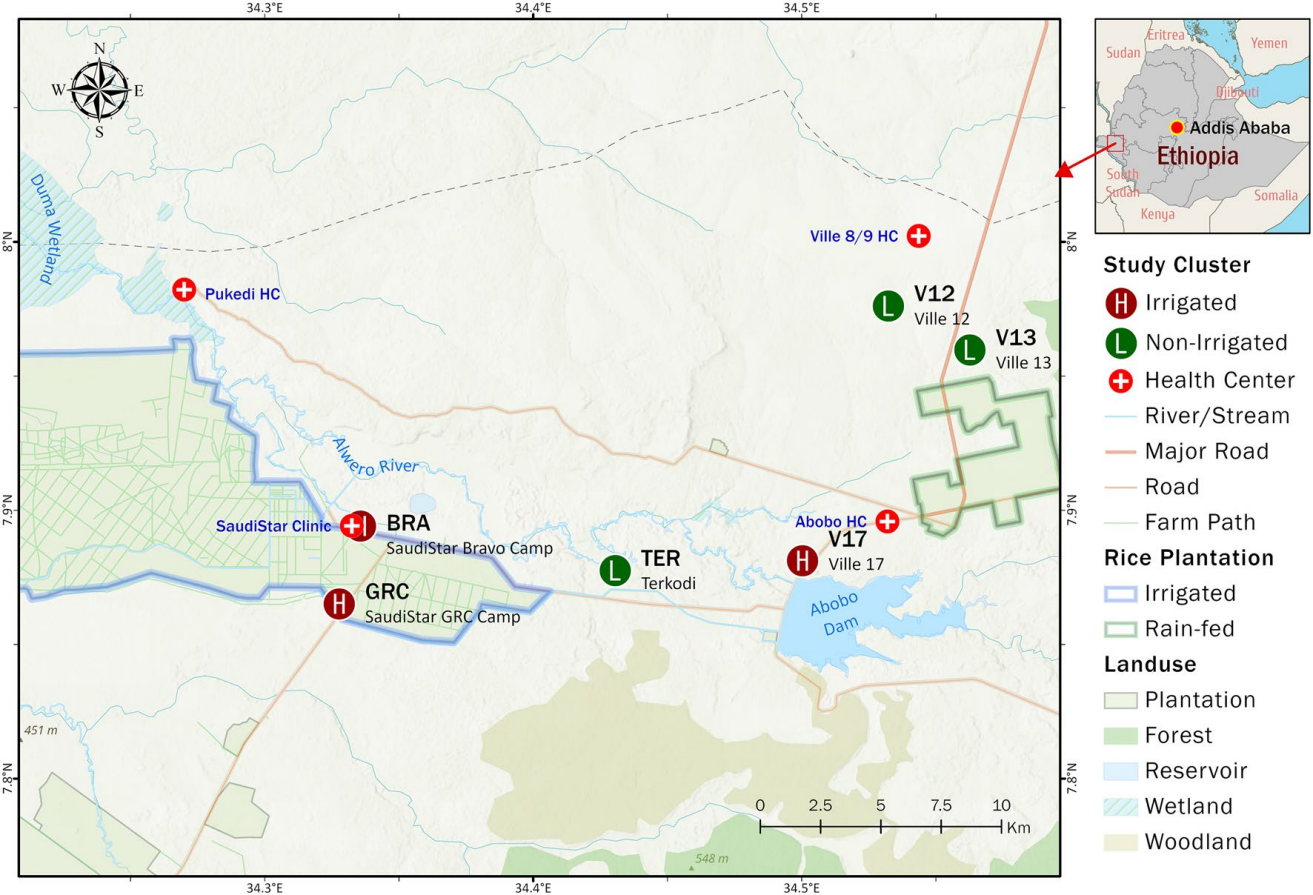
Map of study area

The study site was classified into different clusters. Each cluster was georeferenced to its geographic centroid. Spatial coordinates were used to map the clusters with important landmarks to facilitate identification of clusters by the research team. The clusters were grouped into nearby and faraway mainly depending on their proximity to the rice irrigation scheme (Fig. 1). Clusters within 3 km radius from the irrigation scheme were classified as nearby clusters, while those located 6–10 km from the irrigation site were grouped as faraway clusters (considering flight range of the local mosquito vector population). Of the total 21 clusters identified in the district, six clusters (three from nearby and three from faraway clusters) were purposively selected for the study. A cluster is an area with radius ranging from 250 to 500 m and with 100–250 households. Malaria vector control interventions were implemented in the district using indoor residual spraying and insecticide-treated nets, similar to other malaria endemic areas in Ethiopia. The vector control interventions being implemented were similar in each cluster.

### Entomological study

Adult mosquitoes were collected from all the selected clusters in three rounds in 2018. Centers for Disease Control and Prevention (CDC) light traps (LTs) (John W. Hock Ltd., Gainesville, FL, USA) were used to collect adult mosquitoes during the three survey rounds (February, August and November 2018) from all six clusters. Moreover, during the November survey, mosquitoes were also collected using human landing catches (HLCs) and pyrethrum spray catches (PSCs) from one nearby cluster (Bravo) and one faraway cluster (Mender 13) besides the CDC LT collections.

The CDC LTs were set both indoors (inside bed room on roof support or ceiling) and outdoors (5–8 m from the house where the CDC LT was set indoors) in seven selected houses in each cluster. Mosquitoes were collected from 18:00 to 06:00 from each house for two consecutive nights in the seven selected houses per cluster. The CDC LTs were hung indoors near an occupied bed or mattress protected by bed net, approximately 1.5 m from the ground. The collection bags attached to each of the CDC LTs were labeled. The research team removed the bags from each trap early in the morning from 6:00 to 6:30 am. The CDC LT collection bags were transported to the field entomology lab to sort and identify mosquitoes.

HLC was conducted for two nights in two houses from each of the two clusters in November 2018. Thus, mosquito collection was conducted for a total of eight person-nights (four person-nights in each of the two clusters). Four experienced volunteers (two teams of two people) were involved in mosquito collection using HLC each night switching between indoors and outdoors each hour. The collection was carried out from 18:00 to 06:00 with the mosquitoes captured each hour being kept in separate labeled paper cups. The mosquitoes were collected using aspirator and torch. Volunteers were provided with prophylaxis (mefloquine) as per the national malaria treatment guidelines [1].

Indoor resting anopheline mosquitoes were collected using PSC from 20 houses each in the Bravo (nearby) and Mender 13 (faraway) clusters. Prior to conducting the PSC, human occupants and animals (where present) were evacuated, and food items were removed from each house. White sheets were spread to fully cover the floor. After closing doors and windows, one of the collectors sprayed aerosol insecticide (Baygon, SC Johnson & Son Inc., Racine, WI, USA) in the room. Fifteen minutes after spraying, the sheets were carefully removed from the house and taken outside to look for knocked-down mosquitoes. Mosquitoes were then transferred to petri dishes using forceps for morphological identification. The PSC was conducted earlier in the morning from 06:00 to 08:00.

The collected anopheline mosquitoes were morphologically identified to species level using standard taxonomic keys [22]. The identified mosquitoes were preserved individually in labeled Eppendorf tubes over silica gel for further processing (information on the label included morphological ID and site, date and method of collection).

### Sporozoite ELISA

Sporozoite infection of the female anopheline mosquitoes was tested using circumsporozoite protein (CSP) enzyme-linked immunosorbent assay (ELISA) following an established method [23]. In brief, the head and thorax of the dried anopheline mosquitoes were cut from the abdomen transversely. A pool of 10 anopheline mosquitoes (the head and thorax portion) were ground in a 1.5 ml Eppendorf tube using a pestle and mortar, and thoroughly homogenized in 50 µl grinding buffer. The pestle was rinsed with grinding buffer, making a total volume of 250 µl homogenate of each pool. It was immediately tested for *Plasmodium falciparum*, *P. vivax*-247 and *P. vivax*-210 CSPs as follows: labeled 96-well plates were coated with the respective monoclonal antibodies (mAbs). After incubation for 30 min at room temperature, the solution was removed and blocking buffer added to each well. Following 1 h of incubation, the contents of the wells were discarded in a sink by rapidly turning the plates upside down, and 50 µl negative control, positive control and samples was loaded into the respective wells. The plates were covered and incubated for 2 h. The plates were washed using Tween-20, and peroxidase-labeled conjugate solutions were added and incubated for 1 h. After washing the plates, ABTS (2,2′-azinobis [3-ethylbenzothiazoline-6-sulfonic acid]-diammonium salt) substrate was added to each well and incubated for 30 min. The plates were read using an ELISA reader at 405 nm. Similar anopheline mosquito species collected by a similar method from the same cluster were pooled and analyzed.

### Molecular identification of the *An. gambiae* complex

Sub-samples (*n*  =  120) of *An. gambiae* s.l. were randomly selected and analyzed using species-specific polymerase chain reaction (PCR) following the method reported by Scott et al. [24]. The specimens were sampled from each cluster including both the nearby and faraway clusters and for each trapping method. Genomic DNA was extracted from the legs and wings of specimens using the Qiagen DNA extraction kit. A cocktail of three primers specific for *An. arabiensis* (AR: 5′AAGTGTCCTTCTCCATCCTA3′), *An. gambiae* sensu stricto (GA: 5′ CTGGTTTGGTCGGCACGTTT 3′) and *An. quadriannulatus* species B (*An. amharicus*) (QD: 5′ AGTGTCCAATGTCTGTGAAG 3′), and a universal primer common to *An. gambiae* (UN: 5′ GTGTGCCCCTTCCTCGATGT 3′) were used. The PCR reaction mix (20 µl) contained 1 µl of genomic DNA, 0.5 µl of each primer and 10 µl of DreamTaq PCR Master Mix (2×) (Thermo Scientific). The PCR reaction conditions were as follows: 35 cycles of denaturation at 94 °C for 30 s, annealing at 50 °C for 30 s, and extension at 72 °C for 45 s. The final cycle was followed by extension at 72 °C for 6 min. The PCR products were visualized on 2% agarose gel after staining with ethidium bromide. The molecular analysis (both CSP-ELISA and PCR) was performed in the Molecular Biology Laboratory of Tropical and Infectious Diseases Research Centre (TIDRC), Jimma University. Lab-maintained *An. arabiensis* from TIDRC was used as a positive control.

### Data analysis

Data were entered and cleaned using Microsoft Excel 2010 and analyzed using SPSS version 20.0 software (IBM Corp., Armonk, NY, USA). Descriptive statistics were used to calculate the sporozoite rate (SR), entomological inoculation rate (EIR) and vector density. The SR was calculated for each anopheline species by the method described by Charlwood et al. [25] using the equation $P_{t} = 1 - \exp \left[ {\frac{{\ln \left( {1 - P_{o} } \right)}}{n}} \right],$ where *P*_*t*_  =  true SR, *P*_*o*_  =  observed proportion of positive pools and *n*  =  pool size. The monthly EIR of HLC and CDC LT-collected anopheline mosquitoes was calculated for each species segregated by cluster of collection. From HLC collections it was calculated as the product of the SR and human biting rate (HBR). The HBR was estimated by dividing the total number of each anopheline species by the total person-nights of collection [26]. The EIR from the CDC LT collections for each *Anopheles* species was obtained using the following formula: 1.605 × SR from CDC LT collections × average no. of each *Anopheles* per trap × no. of days per month [27]. Differences in anopheline catches between indoor and outdoor sampling in the same risk area and difference in pooled indoor and outdoor catches between the nearby and faraway clusters were compared using *t-*tests from log-transformed data. Multiple regression analysis was utilized to determine factors independently associated with anopheline mosquito density. Differences in biting rates between indoors and outdoors in the same risk area and differences in biting rates (pooled indoor and outdoor) between the nearby and faraway clusters were compared using Chi-square tests.

## Results

### Species composition

**Fig. 2:**
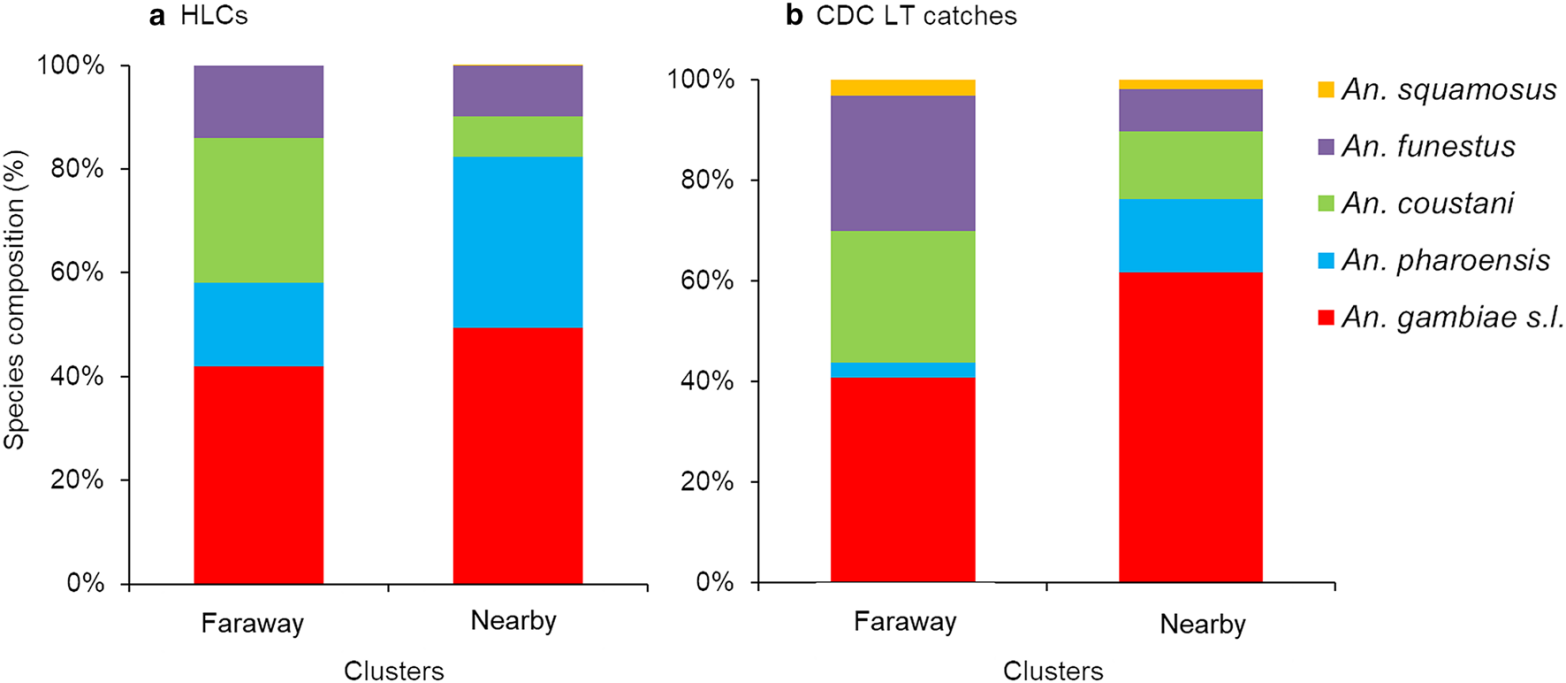
Anopheline mosquito species composition in nearby and faraway clusters in Gambella, Ethiopia, 2018. **a** Anopheline mosquito species collected using HLCs; **b** Anopheline mosquito species collected using CDC light traps

A total of 4319 female anopheline mosquitoes were collected from the six clusters. *Anopheles gambiae* s.l., *An. pharoensis*, *An. coustani* group, *An. funestus* group, and *An. squamosus* comprised 56.5%, 15.7%, 14.4%, 11.7% and 1.6% of the total collections, respectively. Overall, 84.5% and 15.5% of the anopheline mosquitoes were collected from the nearby and faraway clusters, respectively. The anopheline species collected using HLC and CDC LTs are shown in Fig. 2.

Of the 120 subsamples of *An. gambiae* s.l. tested using PCR, 86.7% (104/120) were successfully amplified and all belonged to *An. arabiensis*. The number of each anopheline species and their proportion by risk level is shown in Table 1.

**Table 1:** Abundance of *Anopheles* mosquito species by location of collection and cluster in Gambella, Ethiopia, 2018

Cluster	Collection method	Environment	Anopheline species				
			*An. gambiae* s.l.	*An. funestus*	*An. pharoensis*	*An. coustani*	*An. squamosus*
Nearby	CDC light trap	Indoor	1147 (31.4)	67 (1.8)	207 (5.7)	116 (3.2)	25 (0.7)
		Outdoor	675 (18.5)	185 (5.1)	221 (6.1)	282 (7.7)	27 (0.7)
	HLC	Indoor	106 (2.9)	18 (0.5)	91 (2.5)	21 (0.6)	0 (0.0)
		Outdoor	229 (6.3)	48 (1.3)	135 (3.7)	31 (0.8)	0 (0.0)
	PSC	Indoor	4 (0.1)	14 (0.4)	0 (0.0)	0 (0.0)	0 (0.0)
		Subtotal	2161 (59.2)	332 (9.1)	654 (17.9)	450 (12.3)	52 (1.4)
Faraway	CDC light trap	Indoor	160 (23.9)	99 (14.8)	8 (1.2)	39 (5.8)	2 (0.3)
		Outdoor	87 (13.0)	64 (9.6)	10 (1.5)	121 (18.1)	17 (2.5)
	HLC	Indoor	7 (1.0)	1 (0.1)	3 (0.4)	4 (0.6)	0 (0.0)
		Outdoor	12 (1.8)	6 (0.9)	5 (0.7)	10 (1.5)	0 (0.0)
	PSC	Indoor	12 (1.8)	3 (0.4)	0 (0.0)	0 (0.0)	0 (0.0)
		Subtotal	278 (41.5)	173 (25.8)	26 (3.9)	174 (26.0)	19 (2.8)

Numbers in brackets indicate percent calculated out of total number of anopheline mosquitoes in each risk level

*HLC* human landing catch; *PSC* pyrethrum spray catch

### Anopheline mosquito density

**Fig. 3:**
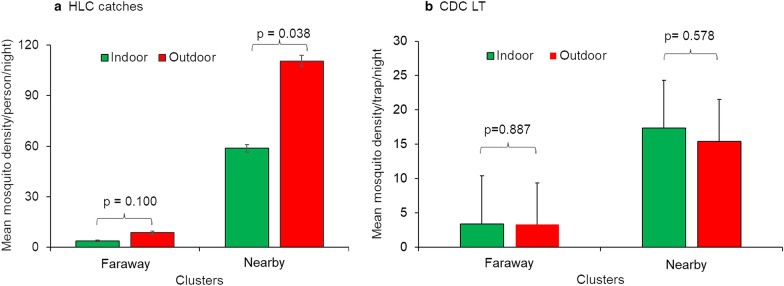
Mean density of anopheline mosquitoes in nearby and faraway clusters in Gambella, Ethiopia, 2018. **a** Anopheline mosquito density collected using HLCs; **b** Anopheline mosquito density collected using CDC LTs

The density of anopheline mosquitoes collected using HLC and CDC LTs in the nearby and faraway clusters is presented in Fig. 3. The density of mosquitoes collected by HLC was significantly higher in the nearby clusters (84.6  ±  14.9 mosquitoes/person/night) than in the faraway clusters (6.3  ±  1.3 mosquitoes/person/night) [*t*_(8)_  =  6.5, *P*  =  0.001]. Similarly, the density of anopheline mosquitoes collected using CDC LTs in the nearby clusters (11.71  ±  1.2 mosquitoes/trap/night) was significantly higher than those collected in the faraway clusters (2.41  ±  0.25 mosquitoes/trap/night) [*t*_(271.97)_  =  7.446, *P*  <  0.0001]. The density of mosquitoes collected in November using HLC (45.4 mosquitoes/person/night) was markedly higher than those collected using CDC LTs (2.4 mosquitoes/trap/night). The density of anopheline mosquitoes collected from each cluster using the different collection methods is shown in Additional file 1: Table S1.

The difference in the density of indoor-collected anopheline mosquitoes in the nearby clusters (12.4  ±  1.9 mosquitoes/trap/night) was significantly higher than that of the indoor collections from the faraway clusters (2.44  ±  0.4 mosquitoes/trap/night) (*P*  <  0.0001). Likewise, the difference in the density of outdoor-collected anopheline mosquitoes in the nearby clusters (11.03  ±  1.5 mosquitoes/trap/night) was significantly higher than those collected from the faraway clusters (2.37  ±  0.4 mosquitoes/trap/night) (*P*  <  0.0001). After controlling for the effects of venue of collection and date, season of collection (*P*  <  0.0001) and risk level (*P*  <  0.0001) were found to have significant effects on the density of anopheline mosquitoes collected using CDC LTs.

Results of mosquito analysis by species showed significantly higher density of *An. gambiae* s.l. [*t*_(261.144)_  =  6.568, *P*  <  0.0001], *An. coustani* group [*t*_(357.072)_  =  4.136, *P*  <  0.0001], *An. pharoensis* [*t*_(254.647)_  =  7.636, *P*  <  0.0001], *An. funestus* group [*t*_(432.036)_  =  2.519, *P*  =  0.012] and *An. squamosus* [*t*_(352.98)_  =  2.535, *P*  =  0.012] collected from nearby clusters compared to faraway clusters. However, there was no significant difference in the overall anopheline density between indoor and outdoor CDC LT catches [*t*_(502)_  =  0.546, *P*  =  0.586]. The density of *An. gambiae* s.l. collected using outdoor CDC LTs was significantly higher than that in the indoor catches in both the nearby and faraway clusters (*P*  <  0.05). Moreover, the density of *An. coustani* collected using outdoor CDC LTs in the faraway clusters was significantly higher than the density in the indoor collections (*P*  <  0.05). Significantly higher density of *An. coustani* was also obtained in outdoor HLC compared to the indoor collections in the nearby clusters (*P*  <  0.05).

The highest density of anopheline mosquitoes was observed in August CDC LT collections [16.4 mosquitoes/trap/night, *F*_(2, 2517)_  =  116.453, *P * <  0.0001]. Pairwise comparisons of the mosquito density showed that the anopheline density in August (16.4 mosquitoes/trap/night) was significantly higher than the density in November and February (2.4 mosquitoes/trap/night each) (*P*  <  0.0001).

### Anopheline mosquito biting activity

**Fig. 4:**
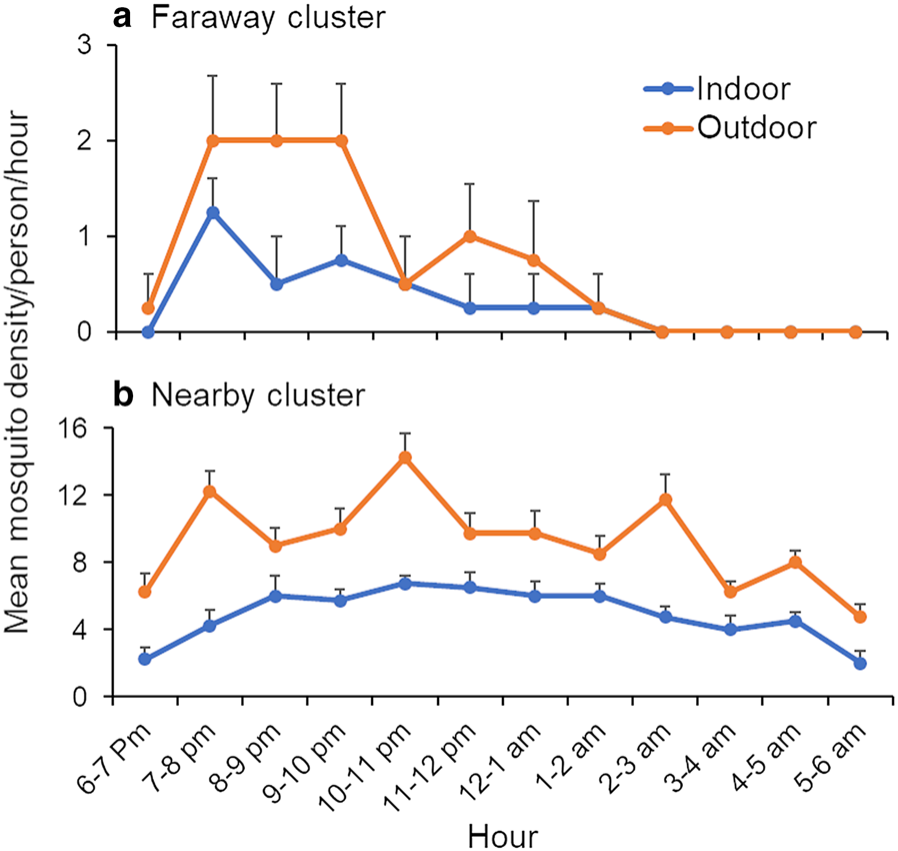
Biting activity of anopheline mosquitoes collected indoors and outdoors in nearby and faraway clusters in Gambella, Ethiopia, 2018. **a** Biting activity of anopheline mosquitoes in the faraway cluster; **b** Biting activity of anopheline mosquitoes in the nearby cluster

In the faraway clusters, biting occurred mainly between 6:00 pm and 2:00 am, with peak activity between 7:00 and 9:00 pm (Fig. 4a), and biting activity was similar between indoors and outdoors (*χ*^2^  =  2.82, *df*  =  7, *P*  =  0.9015). In contrast, in the nearby clusters, mosquito biting activity occurred throughout the night from 6:00 pm to 6:00 am, although there was a slightly higher biting period between 8:00 pm and 2:00 am (Fig. 4b), and biting patterns were similar between indoors and outdoors (*χ*^2^  =  8.39, *df*  =  11, *P*  =  0.6781). The biting patterns were different between the nearby and faraway clusters (*χ*^2^  =  41.18, *df*  =  11, *P*  <  0.0001).

**Fig. 5:**
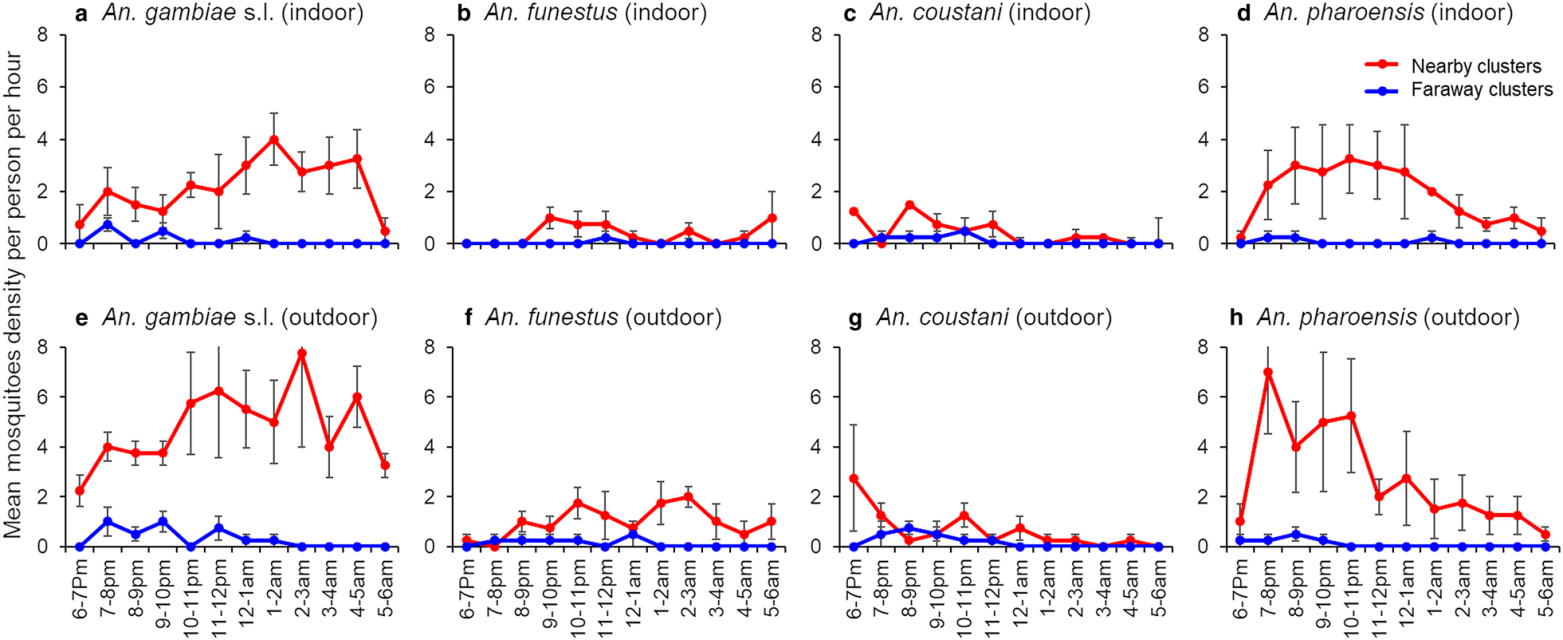
Indoor and outdoor biting activity of anopheline mosquito species from nearby and faraway clusters in Gambella, Ethiopia, 2018. Indoor HLC from nearby and faraway clusters of **a** *An. gambiae* s.l.; **b** *An. funestus*; **c** *An. coustani*; **d** *An. pharoensis*. Outdoor HLC from nearby and faraway clusters of **a** *An. gambiae* s.l.; **b** *An. funestus*; **c** *An. coustani*; **d** *An. pharoensis*

Indoor and outdoor biting activity of *An. gambiae* s.l. and *An. Pharoensis* was markedly more pronounced in the nearby than faraway clusters. Biting activity occurred throughout night for both *An. gambiae* s.l. and *An. pharoensis* in the nearby clusters (Fig. 5). Noticeably higher outdoor biting activity was recorded in *An. Funestus *in the nearby clusters compared to the faraway clusters.

### Entomological inoculation rate

Four of the five anopheline species from the nearby clusters were positive for CSP, while a single specimen of *An. coustani* was positive for CSP from the faraway clusters. The overall SR of the different anopheline species collected from the nearby clusters was 10-fold higher than that of the faraway clusters. The overall monthly *P. falciparum* and *P. vivax* EIR of the anopheline species collected using the different methods from the nearby clusters was 7.6 and 23.5 infective bites/person/month, respectively. In contrast, the corresponding monthly *P. falciparum* and *P. vivax* EIR of the anopheline mosquitoes collected from the faraway clusters was zero and 0.2 infective bites/person/month, respectively (Table 2).

**Table 2:** Sporozoite rates and entomological inoculation rates of *Anopheles* mosquito species from nearby and faraway clusters in Gambella, Ethiopia, 2018

Cluster	Method of collection	Species	Total tested	No. of pools positive (SR)	SR			EIR	
					Pf	Pv-210	Pv-247	Pf	Pv
Nearby	CDC light trap	*An. coustani*	398 (43)	4 (0.97)	0	0.48	0.48	0	0.7
		*An. funestus* group	252 (31)	1 (0.33)	0.33	0	0	0.2	0
		*An. gambiae* s.l.	1822 (189)	2 (0.11)	0	0.05	0.05	0	0.4
		*An. pharoensis*	428 (47)	0	0	0	0	0	0
		*An. squamosus*	52 (6)	0	0	0	0	0	0
		Subtotal	2952 (316)	7 (0.22)	0.03	0.1	0.1	0.2	1.1
	HLC	*An. coustani*	52 (6)	1 (1.81)	0	1.81	0	0	3.5
		*An. funestus* group	66 (7)	1 (1.53)	1.53	0	0	3.8	0
		*An. gambiae* s.l.	335 (34)	2 (0.60)	0.3	0	0.3	3.8	3.8
		*An. pharoensis*	226 (23)	4 (1.89)	0	0.9	0.9	0	16.0
		Subtotal	679 (70)	8 (1.21)	0.29	0.44	0.44	7.4	22.4
	PSC	*An. gambiae* s.l.	4 (1)	0	0	0	0	0	0
		*An. funestus* group	14 (2)	0	0	0	0	0	0
		Subtotal	18 (3)	0	0	0	0	0	0
Faraway	CDC light trap	*An. coustani*	160 (19)	1 (0.54)	0	0.54	0	0	0.2
		*An. funestus* group	163 (19)	0	0	0	0	0	0
		*An. gambiae* s.l.	247 (28)	0	0	0	0	0	0
		*An. pharoensis*	18 (3)	0	0	0	0	0	0
		*An. squamosus*	19 (2)	0	0	0	0	0	0
		Subtotal	607 (71)	1 (0.14)	0	0.14	0	0	0.2
	HLC	*An. coustani*	14 (2)	0	0	0	0	0	0
		*An. funestus* group	7 (1)	0	0	0	0	0	0
		*An. gambiae* s.l.	19 (2)	0	0	0	0	0	0
		*An. pharoensis*	8 (1)	0	0	0	0	0	0
		Subtotal	48 (6)	0	0	0	0	0	0
	PSC	*An. gambiae* s.l.	12 (2)	0	0	0	0	0	0
		*An. funestus* group	3 (1)	0	0	0	0	0	0
		Subtotal	15 (3)	0	0	0	0	0	0

*EIR* entomological inoculation rate per month; *HLC* human landing catch; *PSC* pyrethrum spray catch; *SR* corrected sporozoite rate

## Discussion

The overarching objective of the study was to assess the effect of rice irrigation on the risk of malaria transmission in Gambella, Ethiopia. Accordingly, more than 10-fold higher SR of the anopheline mosquitoes was obtained from the nearby clusters compared to the faraway clusters. Monthly *P. vivax* EIR of *An. coustani* collected from the nearby clusters was nearly 25-fold higher compared to the faraway clusters. None of the other anopheline species collected from the faraway clusters were CSP-positive. These indicate the impact of the irrigation scheme on malaria transmission intensity in the area. The findings show that the risk of malaria transmission was remarkably higher in the irrigation sites compared to the non-irrigation sites. Several other similar studies also documented the effects of irrigation in enhancing malaria transmission [3, 15]. The lower SR in the faraway clusters could also be related to the low density of mosquitoes collected from these clusters. The overall SR of the anopheline mosquitoes collected using HLC was fourfold higher than the SR obtained using CDC LT collections in the nearby clusters. The lower SR from CDC LT collections could be attributed to higher proportion of nulliparous female mosquitoes collected by CDC LTs [28].

Adult anopheline density was significantly higher in the nearby clusters compared to the faraway clusters. This could be attributable to favorable microhabitats formed by the agro-ecosystem, allowing proliferation of a range of anopheline mosquitoes. The higher density of anopheline species observed in the irrigation clusters could also be due to the odor of the rice. Rice odor surrounding rice fields elicits attraction and oviposition in gravid female *An. arabiensis* [29], the dominant anopheline species in the area. Moreover, there could be differences in survivorship of adult *An. arabiensis* in the nearby and faraway clusters [30]. Alterations in the microclimatic environment in the rice irrigated areas resulting from deforestation might also have contributed to the observed higher mosquito density in the irrigated clusters [31]. Immature stages of *An. arabiensis* typically prefer sunlit pools [7], which may be created as a result of deforestation for rice cultivation. This allows the presence of abundant sunlit aquatic habitats to be created, increasing the water temperature, making the habitat conducive for larval development. Increased temperature and relative humidity resulting from deforestation in irrigation areas may also affect the extrinsic incubation period of the malaria parasites in mosquito guts [32], ultimately enhancing transmission.

Outdoor biting by malaria vectors is a huge challenge for malaria control and elimination efforts, and may contribute a substantial number of additional malaria cases after deployment of the indoor-based interventions [33]. In this study, two-thirds of the anophelines collected using HLC were captured outdoors. The density of outdoor host-seeking anopheline mosquitoes was significantly higher in the nearby clusters compared to the faraway clusters. This shows higher risk of outdoor transmission of malaria, which may sustain residual transmission near rice irrigation areas. Moreover, biting activity throughout the night near irrigation areas poses particular risk to individuals involved in nighttime activities and those who may sleep outdoors. The tendency to bite predominantly outdoors earlier in the evening in the faraway clusters may also contribute to residual transmission of malaria in these areas.

Anthropogenic manipulation of water resources, including development of irrigation schemes and hydroelectric dams, is being intensified in Ethiopia and other developing countries to provide food and energy to satisfy the fast-growing population [12, 13]. Although irrigation activities do not necessarily lead to increased malaria transmission [8], surveillance of vector-borne disease in general and malaria in particular is critically required. The observed higher vector density near the rice irrigation scheme could be attributed to favorable breeding habitats created by the rice paddies. As Alwero Dam supplies water to the main irrigation canals, the rice field obtains continuous supply of water, which enables prolonged flooding of the rice fields during dry season.

In Ethiopia, malaria is principally transmitted by *An. arabiensis*, with *An. pharoensis* playing a secondary role and *An. coustani* being a suspected vector. Due to its limited geographical distribution, *An. funestus* also plays a secondary role in malaria transmission in some areas in Ethiopia [1, 2]. This study revealed that four of the five mosquito species collected (with *An. squamosus* being the exception) were CSP-positive, indicating that malaria is likely transmitted by multiple vector species in this area. *An. pharoensis* had the highest EIR, which is not the case in other areas of Ethiopia [34, 35]. Historical data also showed that *An. pharoensis* was found to be parasite-infected in the Gambella area [36]. A recent mosquito infection study using membrane feeding assay documented that *An. pharoensis* was as susceptible as *An. gambiae* s.l. to *P. vivax* infections [37]. Infectivity of *An. pharoensis* in this area is likely due to increase in its longevity associated with microclimate change (increase in relative humidity) in irrigation areas [38]. Repeated gonotrophic cycles are likely in the long-surviving adult vectors, possibly perpetuating malaria transmission. Higher survivorship in other anopheline species in lower altitudes (hotter areas) was documented elsewhere as well [39]. However, it should also be noted that the highest EIR obtained in *An. pharoensis* specimens could also be due to the low number of *An. pharoensis* collected and analyzed.

The effect of WRDPs on the bionomics of anopheline mosquitoes is complex. Limited studies done earlier in Ethiopia documented increased malaria transmission associated with irrigation, focusing on sugar cane and crops other than rice [15, 40]. The impact of rice irrigation on the risk of malaria in Ethiopia has not been explored. Flooded paddy fields may create conducive breeding habitats for a wide range of anopheline mosquitoes, ultimately enhancing malaria transmission intensity in the locality. Contrary to the findings of this study, other investigations have found no impact of rice irrigation on the risk of malaria, and reduced risk in rice irrigated areas has even been reported [5, 8]. Therefore, it appears that the ultimate impact of irrigation schemes on the epidemiology of malaria is not straightforward. The impact of such projects on the risk of malaria partly depends on the level of endemicity of malaria, the type of principal malaria vectors in the area, the ecological setting of the irrigation scheme (whether located in lowland or highland area), the status of vector management, and the economic return obtained by the local community from the irrigation scheme [5, 8, 18]. It should be noted that in this study, mosquitoes were sampled using HLC from one village each from the nearby and faraway clusters during one sampling season. This limited the number of mosquitoes processed from the faraway clusters.

## Conclusions

In conclusion, higher anopheline density was recorded in the irrigation areas compared to the non-irrigation areas. The EIRs of the anopheline mosquitoes were also higher in clusters near the irrigation scheme. Multiple anopheline mosquito species including secondary and suspected malaria vector species were found sporozoite-positive in the irrigation sites compared to non-irrigation sites. Hence, the irrigation scheme in the study area could increase the risk of malaria transmission in communities residing in proximity to the irrigation sites. WRDPs including irrigation schemes should be critically monitored for their potential effect on transmission of vector-borne diseases in general and malaria in particular. Further studies are required to better understand the epidemiology of malaria in the area.

## Supplementary Information

**Additional file 1: Table S1. ***Anopheles* mosquito density and sporozoite rate by method of collection and study cluster in Gambella, Ethiopia (2018).

## Data Availability

The data supporting the results reported in this article are included within the article and its supplementary file.

## Abbreviations

CDC LT: Centers for Disease Control and Prevention light trap
CSP: Circumsporozoite protein
EIR: Entomological inoculation rate
ELISA: Enzyme-linked immunosorbent assay
HLC: Human landing catch
PCR: Polymerase chain reaction
PSC: Pyrethrum spray catch
SR: Sporozoite rate
WRDP: Water resource development project
