# Correction to: The effect of irrigation on malaria vector bionomics and transmission intensity in western Ethiopia

**DOI:** 10.1186/s13071-021-05083-9

**Published:** 2021-11-18

**Authors:** Werissaw Haileselassie, Endalew Zemene, Ming-Chieh Lee, Daibin Zhong, Guofa Zhou, Behailu Taye, Alemayehu Dagne, Wakgari Deressa, James W. Kazura, Guiyun Yan, Delenasaw Yewhalaw

**Affiliations:** 1 School of Public Health, College of Health Sciences, Addis Ababa University, Addis Ababa, Ethiopia; 2 School of Medical Laboratory Sciences, Institute of Health, Jimma University, Jimma, Ethiopia; 3 Program in Public Health, College of Health Sciences, University of California at Irvine, Irvine, CA 92697 USA; 4 Department of Biology, Faculty of Natural and Computational Science, Mettu University, Mettu, Ethiopia; 5 Center for Global Health and Disease, Case Western Reserve University, Cleveland, OH 44106 USA; 6 Tropical and Infectious Diseases Research Centre, Jimma University, Jimma, Ethiopia

## Correction to: Parasites Vectors (2021) 14: 516 https://doi.org/10.1186/s13071-021-04993-y

Following publication of the original article [1], it was brought to our attention that incorrect grant numbers had been provided in the funding declaration of the article.

The following grant numbers had been provided: “U19 AI089672 and D43 TW009527”.

However, the correct grant numbers are as follows: “U19 AI129326 and D43 TW001505”.

The original article has been updated to correct these numbers.

The authors thank you for reading this correction and apologize for any inconvenience caused.
