# Supplementary material for: The effect of irrigation on malaria vector bionomics and transmission intensity in western Ethiopia
Source: Parasit Vectors. 2021 Oct 7;14:516. doi: 10.1186/s13071-021-04993-y (PMC8500124; doi:10.1186/s13071-021-04993-y)
Supplement: Supplementary file 1 — Additional file 1: Table S1. Anopheles mosquito density and sporozoite rate by method of collection and study cluster in Gambella, Ethiopia (2018). [file 13071_2021_4993_MOESM1_ESM.docx]

**Additional file 1: Table S1.** *Anopheles* mosquito density and sporozoite rate by method of collection and study cluster in Gambella, Ethiopia (2018)

Key: HLC: Human landing catch, PSC: Pyrethrum spray catch, SR: Sporozoite rate

*Mosquito/trap/night (CDC Light trap), mosquito/person/night (HLC) and mosquito/house (PSC)

| Method of collection | Cluster | # Mosquitoes collected | Mosquito density* | Pf SR | Pv SR |
| --- | --- | --- | --- | --- | --- |
| CDC Light trap | Bravo | 1916 | 22.8 | 0.05 | 0.21 |
|  | GRC | 785 | 9.3 | 0 | 0.13 |
|  | Village 17 | 251 | 3.0 | 0 | 0.40 |
|  | Terkudy | 145 | 1.7 | 0 | 0.69 |
|  | Village 12 | 20 | 0.2 | 0 | 0 |
|  | Village 13 | 442 | 5.3 | 0 | 0 |
| HLC | Bravo | 677 | 84.6 | 0.30 | 0.88 |
|  | Village 13 | 50 | 6.3 | 0 | 0 |
| PSC | Bravo | 18 | 0.9 | 0 | 0 |
|  | Village 13 | 15 | 0.6 | 0 | 0 |
